# Pre-arrival OCC decision support for aircraft turnaround coordination: a human-in-the-loop architecture and scenario-based evaluation

**DOI:** 10.3389/frai.2026.1977034

**Published:** 2026-10-19

**Authors:** Parth Yogeshbhai Purohit, Thomas Feuerle, Peter Hecker

**Affiliations:** Institut für Flugführung, Technische Universität Braunschweig, Braunschweig, Germany

**Keywords:** aircraft turnaround, bottleneck forecasting, decision support, human-in-the-loop, inventory readiness, operations control center, predictive maintenance, scenario-based evaluation

## Abstract

Aircraft turnaround coordination depends on maintenance, catering, passenger-assistance, gate, and bottleneck information that may become available before arrival but is often handled through fragmented workflows. This paper presents a human-in-the-loop Operations Control Center (OCC) decision-support architecture for pre-arrival aircraft turnaround coordination. The architecture integrates component-aware maintenance risk indicators, service-area inventory readiness, process-class bottleneck forecasting, passenger-assistance readiness logic, and a transparent rule-based fusion layer that converts heterogeneous signals into readiness scores, alert levels, and operator-facing action queues. The evaluation is deliberately framed as controlled synthetic scenario analysis rather than airline deployment validation. Existing module-level holdout results are combined with a deterministic scenario sweep of 1,000 synthetic inbound cases across six operational regimes, monotonicity and sanity checks, and ablation and baseline comparisons. The scenario sweep produced mean readiness 47.4, median readiness 50.6, 370 Critical cases, 284 Priority cases, and 188 Routine cases. Ablations show that removing maintenance influence increased mean readiness by 13.7 points, while removing bottleneck influence increased mean readiness by 10.0 points, indicating that the fusion layer materially changes prioritization under controlled stress conditions. Two maintenance-related monotonicity checks did not strictly pass because small local readiness increases occurred at low malfunction levels before urgency thresholds changed; these failures are treated as limitations of the current model-fusion interaction rather than hidden. The work contributes a reproducible OCC decision-support architecture and scenario-evaluation package, while explicitly preserving the limitations that no airline operational data, OCC user study, deployment, certification, or measured operational savings are claimed.

## Highlights

- Pre-arrival operations control support is tested for aircraft turnaround.
- Maintenance, inventory, bottleneck, and assistance signals are fused.
- One thousand synthetic cases cover six operational regimes.
- Signal-removal tests show how inputs change readiness and actions.
- Limitations state no airline data, deployment, user study, or savings claim.

## 1 Introduction

Aircraft turnaround operations are managed under tight time constraints and require coordination across maintenance, catering, cleaning, boarding, refueling, passenger assistance, gate management, and airline operations control. Information relevant to these processes can be available before arrival, but it is often distributed across separate operational or analytical views. The practical problem is therefore not only whether individual prediction models can be trained, but how heterogeneous pre-arrival signals can be converted into a form that supports OCC coordination before the aircraft reaches the stand.

This study is framed as an air transport operations contribution. The focus is pre-arrival decision support for turnaround coordination, not a generic intelligent-systems contribution. The architecture remains human-in-the-loop: it supports operator review through readiness scores, alert levels, provenance labels, and action queues, but it does not automate dispatch or claim certified operational control. This work uses synthetic but operationally grounded data because no real airline, airport, or station-operation dataset is available in the current project package. That boundary is central to the paper. The evaluation therefore asks whether the architecture behaves coherently and differentiates operational regimes under controlled scenarios. It does not ask whether the system is validated for airline deployment.

## 2 Related work

### 2.1 OCC decision support and disruption management

Airline Operations Control Centers coordinate time-critical decisions across aircraft, crews, gates, passengers, and downstream rotations. Classical OCC and disruption-management literature shows that these decisions combine schedule recovery, resource coordination, and human judgment rather than a single optimization objective (Clarke, 1998; Clausen et al., 2010; Hassan et al., 2021; Su et al., 2021). Naturalistic and decision-support studies further motivate systems that preserve operator review instead of replacing OCC staff with automated control (Vink et al., 2020; Fogaca et al., 2022).

### 2.2 Turnaround coordination and ground handling

Aircraft turnaround research treats the stand-side process as a tightly coupled operational system. Work on turnaround management, digital-twin support, apron-resource planning, ground-resource planning, schedule recovery, and ground-handling prediction shows that local process delays can affect broader airline and airport performance (Makhloof et al., 2014; Evler et al., 2021a,b, 2022; Conde et al., 2022; Guimarans and Padron, 2022; Gok et al., 2023; Wu et al., 2023). This paper builds on that perspective by focusing on pre-arrival coordination signals that can change preparation before the aircraft reaches the stand.

### 2.3 Aviation ML for delay and resource prediction

Aviation machine-learning studies have addressed arrival-time prediction, delay classification, resource planning, smart-aviation applications, and ground-operation forecasting (Basturk and Cetek, 2021; Zoutendijk and Mitici, 2021; Sahadevan et al., 2023; Jiang et al., 2023; Okwir et al., 2025; Kicinger et al., 2025; Wandelt et al., 2025). However, a collection of separate predictive tasks does not automatically create an OCC workflow. The contribution here is therefore not a new prediction algorithm; it is the operational integration of heterogeneous predictive and rule-based signals into one pre-arrival advisory process.

### 2.4 Predictive maintenance, service readiness, and assistance

Predictive-maintenance studies motivate maintenance-risk indicators for aviation decision support (Dangut et al., 2022, 2023; Lee and Mitici, 2023; Zeng and Liang, 2023; Hakami, 2024; Kabashkin and Shoshin, 2024). Adjacent work on food-service and demand forecasting is relevant to service-readiness reasoning, but it should not be interpreted as evidence for item-wise airline trolley-demand prediction in this paper (Malefors et al., 2022; Rodrigues et al., 2024). Passenger-assistance and accessibility studies motivate explicit assistance-readiness handling, while the implemented system treats assistance as rule-based readiness support rather than localization machine learning (Gotti et al., 2024; Lee et al., 2025).

### 2.5 Human-in-the-loop and synthetic scenario evaluation

Human-in-the-loop AI and explainability work supports transparent advisory systems where model outputs remain reviewable by human operators (Xie et al., 2021; Mosqueira-Rey et al., 2023; Vaccaro et al., 2024). Synthetic-data and scenario-evaluation studies provide a methodological basis for controlled evaluation when operational data are not available, but they do not remove the need for future airline data and user studies (Ardebili et al., 2023; de Frutos Carro et al., 2024; Stefani et al., 2025). This paper therefore uses synthetic scenarios to evaluate architecture behavior, not to claim field validity. Across these streams, the open gap is the connection between module-level analytics and pre-arrival OCC coordination. Maintenance, inventory, bottleneck, and assistance-readiness signals are not treated as separate dashboards. They are fused into one operator-facing advisory workflow, with explicit labels distinguishing trained inference, rule-based readiness logic, and synthetic scenario simulation.

### 2.6 Safety science, complexity, and risk governance

Decision support introduced into a safety-critical coordination environment is not only a technical artifact; it becomes part of the socio-technical system it observes. Three strands of safety science frame this. First, complexity-informed accident theory argues that failure in tightly coupled systems is an emergent property of interactions rather than a linear chain traceable to a single broken component, and that “Newtonian” explanations—single cause, time-reversible, fully foreseeable—systematically misdescribe how complex systems fail (Dekker et al., 2011). Second, work on organizational risk argues that safety performance in complex operational and business environments is governed less by individual workplace hazards than by organizational structures, incentives, communication patterns, and governance, so that a purely technical intervention can be absorbed, adapted, or defeated by the organization receiving it (Komljenovic et al., 2017). Third, research on major-accident risk warns that sophisticated risk tooling can generate complacency and an illusion of control, in which the visible management of quantified risk creates confidence while un-quantified uncertainties and systemic vulnerabilities persist (Årstad and Aven, 2017).

These perspectives bear directly on a pre-arrival OCC advisory layer. A readiness score is a quantification that will compete for operator attention with signals that are not scored; an action queue is an intervention in an existing pattern of coordination; and an alert threshold is an organizational rule as much as a numerical one. We therefore treat the architecture in this paper not only as an information-integration problem but as a prospective change to a coordination system, and we return to the complexity, organizational, and complacency implications in Sections 11.2 to 11.5.

## 3 Contributions

This study addresses a specific operational question: which pre-arrival signals most change cross-domain turnaround prioritization, and how can heterogeneous predictive and rule-based signals be fused into a transparent, operator-reviewable advisory workflow before the aircraft reaches the stand?

Against that question, the paper makes the following bounded contributions:

- A pre-arrival OCC decision-support architecture for aircraft turnaround coordination.
- A modular integration of maintenance risk, inventory readiness, bottleneck forecasting, and passenger-assistance readiness signals.
- A transparent fusion/action layer that converts heterogeneous signals into readiness scores, alerts, and action queues for operator review.
- A scenario-based evaluation package with seeded cases, a 1,000-case synthetic sweep, monotonic sanity checks, and ablation/baseline comparisons.
- An evidence-bounded design finding that, under controlled scenarios, maintenance and bottleneck signals dominate the change in fused readiness and operator action selection, indicating where pre-arrival coordination attention is most leveraged.

The contribution is architectural and operational, framed for air transport operations: it shows how fragmented pre-arrival information can be organized into a single transparent OCC coordination workflow. It is not a claim of a new AI algorithm, airline deployment, or field-validated performance.

## 4 System architecture

Figure 1 provides an overview of the integrated decision-support architecture, showing the progression from pre-arrival inputs through analytical modules and fusion to operator review, while Figure 2 details the specific module-to-fusion workflow, emphasizing how the different predictive and rule-based modules feed into the transparent OCC fusion layer.

**Figure 1:**
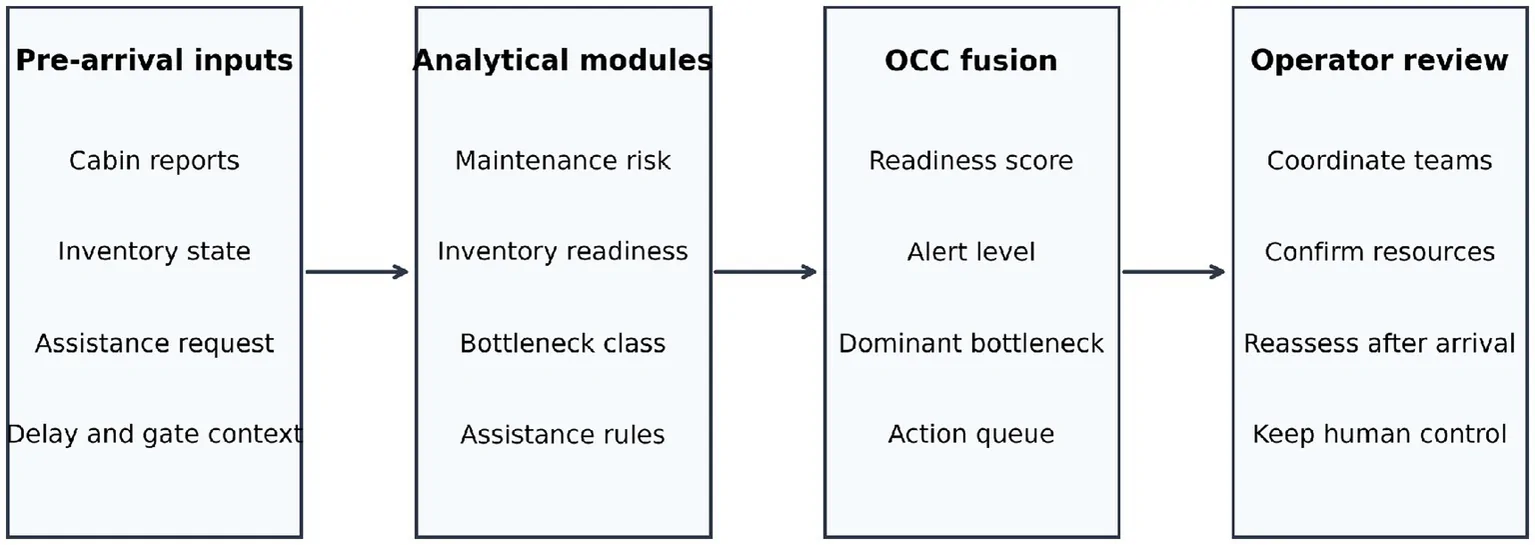
Integrated OCC decision-support architecture for pre-arrival turnaround coordination.

**Figure 2:**
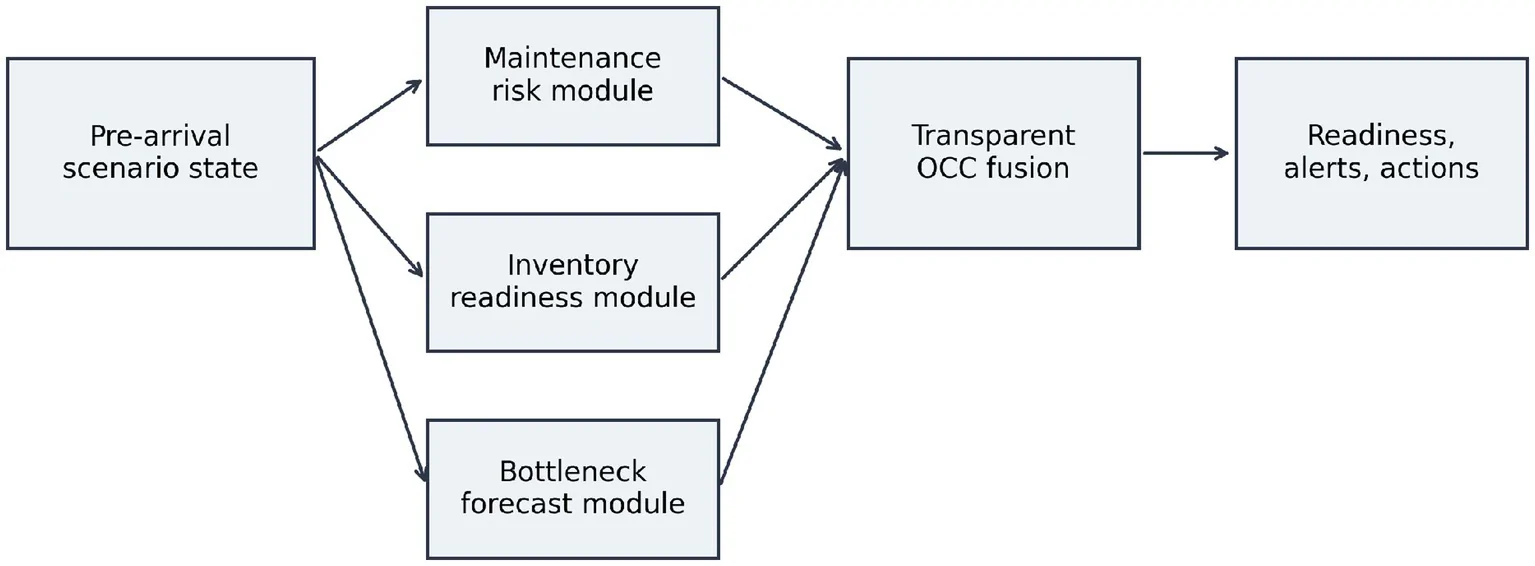
Module-to-fusion workflow for pre-arrival OCC decision support.

The architecture has four layers. The input layer represents pre-arrival aircraft and turnaround state, including passenger load, arrival delay, gate congestion, malfunction count and severity, trolley availability, inventory shortage indicators, assistance requests, weather disturbance, and refueling requirement. The analytics layer contains trained local modules for maintenance, inventory, and bottleneck prediction. Passenger assistance is treated as rule-based readiness support, not localization machine learning. The fusion layer combines module outputs and scenario state into readiness, criticality, alerts, and action queues. The presentation layer exposes the result in an operator-facing OCC interface.

## 5 Module design and evidence boundary

Table 1 provides a consolidated view of the module-level model selection, validation metrics, and output granularity for the predictive maintenance, inventory intelligence, and bottleneck prediction modules. As detailed in the table, the architecture integrates diverse model types—ranging from Logistic Regression for classification tasks to Gradient Boosting for regression—while explicitly maintaining the evidence boundaries associated with each. These boundaries reiterate the synthetic-data constraints of the current study, noting that the outputs support architectural reasoning rather than certified maintenance planning or field-validated operational performance.

**Table 1:** Module-level model summary and evidence boundary.

Module	Task	Selected model	Validation metric	Output granularity	Evidence boundary
Predictive maintenance	Maintenance failure risk	Logistic regression	ROC-AUC 0.740	Component-aware features; flight-scenario risk output	Synthetic-data support only; not certified maintenance planning
Predictive maintenance	Maintenance urgency class	Logistic regression	Macro-*F*1 0.857; ROC-AUC OVR 0.976	Component-aware features; flight-scenario urgency label	Synthetic-data support only; not certified maintenance planning
Predictive maintenance	Remaining-flights proxy	Gradient boosting regressor	RMSE 4.020; *R*^2^ 0.925	Flight-scenario remaining-flights proxy	Synthetic-data support only; not certified maintenance planning
Inventory intelligence	Inventory risk level	Logistic regression	Macro-*F*1 0.748; ROC-AUC OVR 0.903	Service-area, trolley-level, and flight-level inventory-risk support	Synthetic service-readiness risk; not item-wise demand or airline validation
Bottleneck prediction	Bottleneck class	Logistic regression	Macro-*F*1 0.696; ROC-AUC OVR 0.941	Process-class bottleneck probabilities	Synthetic process-class probabilities; not field duration validation

The maintenance module is component-aware in its features and explanation logic, but the OCC interface summarizes the result at the flight-scenario level. It should not be described as a certified component-wise aircraft-health dashboard. The inventory module supports service-area, trolley-level, and flight-level inventory-risk reasoning; it is not item-wise product demand prediction. The bottleneck module predicts process-class bottleneck probabilities across Maintenance, Catering, Cleaning, Boarding, and Refueling. Passenger assistance is rule-based readiness support.

### 5.1 Formal specification of the fusion layer

The fusion layer is deliberately a transparent, deterministic rule set rather than a learned model. Readiness is computed on a 0–100 scale as a base score reduced by independent, non-negative penalty terms, one per signal family:Inputs
m.urgency_class in {OK, PLAN, SOON, CRITICAL} maintenance urgency (module)
m.failure_prob in [0,1] predicted failure probability (module)
i.shortage_risk in [0,1] inventory shortage risk (module)
i.risk_level in {LOW, ELEVATED, HIGH} inventory class (module)
b.prob[c] in [0,1] for c in {Maintenance, Catering, Cleaning, Boarding, Refueling}
gate_congestion in [0,10] scenario state
arrival_delay in minutes scenario state
assist_request, assist_confirmed in {true,false} scenario state
passenger_load scenario state
Derived
b_max=max_c b.prob[c]
Penalties (all >=0; larger=worse)
P_maint={OK:3, PLAN:10, SOON:18, CRITICAL:30}[m.urgency_class]
P_fail=9 * m.failure_prob
P_inv=11 * i.shortage_risk
P_bneck=14 * b_max
P_cong=1.25 * gate_congestion
P_delay=if arrival_delay <= 15: 0.26 * arrival_delay
else: (0.26*15)+min(14, 0.28 * (arrival_delay - 15)^1.12)
P_delay <− min(P_delay, 18)
P_assist=9 if (assist_request and not assist_confirmed) else 0
Compounding (super-additive escalation)
D=number of severe domains simultaneously active, where a domain is severe if:
maintenance: m.urgency_class=CRITICAL or m.failure_prob >=0.72
inventory: i.risk_level=HIGH or i.shortage_risk >=0.78
bottleneck: b_max >=0.62
margin: gate_congestion >=7.0 or arrival_delay >=20
P_comp={0:0, 1:0, 2:4, 3:7, >=4:10}[D]
Readiness and criticality
R=clip(100 - (P_maint + P_fail + P_inv+P_bneck + P_cong + P_delay + P_assist + P_comp), 5, 99)
C=min(100, (100 - R)+12*m.failure_prob + 8*i.shortage_risk + 13*b_max)
Alert level (disjunctive escalation on either channel)
Critical if C>= 80 or R<= 38
Priority if C>= 61 or R<= 56
Watch if C>= 42 or R<= 74
Routine otherwise
Action queue
A<- empty
maintenance dispatch + spare-part reservation if m.urgency_class in {SOON, CRITICAL}
(P1 if CRITICAL, else P2)
inventory top-up if i.risk_level in {ELEVATED, HIGH}
(P1 if HIGH, else P2)
bottleneck-specific coordination by dominant bottleneck class
(Catering P2, Cleaning P3, Boarding P3,
Refueling P3; plus a P2 boarding-lane item
when dominant=Boarding and load >=170)
assistance pre-positioning if assist_request and not assist_confirmed (P1)
gate buffer allocation if arrival_delay >=15 or gate_congestion >=6 (P2)
cross-domain supervisor coordination if D>= 1 (P1 if D>= 3, else P2)
standing operator-review item always (P4)
deduplicate A on (team, description); sort ascending by priority


*Resolution of conflicting signals*: The scheme is deliberately non-compensatory in the protective direction: every penalty is non-negative, so a benign domain contributes zero or a small penalty but can never offset the penalty contributed by a severe domain. A case with critical maintenance risk and nominal inventory therefore retains the full maintenance penalty; low inventory risk cannot buy back readiness. Conflict is thus resolved by dominance of the most severe contributor rather than by averaging, and simultaneous severity across domains is escalated super-additively through the compounding term. The alert mapping applies the same conservatism by escalating if either the criticality channel or the readiness channel crosses a threshold. Each penalty is retained and displayed individually in the readiness breakdown (Section 10), so an operator can always see which domain drove a score.

*Why a rule-based fusion*: Both learned fusion and formal expert elicitation were considered and rejected for this study, for reasons that are properties of the evidence base rather than preferences. A learned fusion requires labeled OCC decisions—observed operator actions paired with outcomes—and no such dataset was available; fitting a fusion to the same synthetic labels used by the modules would manufacture apparent performance without adding evidence. Formal expert elicitation, for example AHP or Delphi weighting, would require an OCC expert panel that the project could not convene, and the resulting weights would still require validation against real operations before they could be treated as calibrated. A transparent rule set was therefore chosen because it is fully inspectable, attributable per signal, and straightforwardly replaceable: the penalty coefficients above are explicit heuristics, not calibrated parameters, and we report them so that they can be challenged, re-elicited, or replaced by a learned layer once operational decision data exist.

## 6 Scenario-sweep design

Table 2 provides an aggregate summary of the 1,000-case synthetic sweep, including mean readiness scores and alert-level frequencies. The evaluation extends the five seeded demonstration cases with a deterministic scenario sweep. The sweep contains 1,000 synthetic inbound cases generated under six regimes: low-risk routine, maintenance-heavy, inventory-heavy, congestion/delay-heavy, assistance-readiness, and composite multi-risk. A fixed random seed is used so that the inputs, outputs, and figures can be reproduced. Cases are assigned across the six regimes and then evaluated through the existing trained model inference and OCC fusion functions.

**Table 2:** Overall scenario-sweep summary.

Metric	Value
Scenario count	1,000
Mean readiness	47.40
Median readiness	50.65
Mean action queue size	4.60
Routine alerts	188
Watch alerts	158
Priority alerts	284
Critical alerts	370
Maintenance bottleneck count	319
Catering bottleneck count	186
Boarding bottleneck count	286

The generator varies arrival delay, gate congestion, malfunction count, malfunction severity, trolley availability, inventory shortage, passenger load, assistance readiness, weather disturbance, and refueling requirement. Low-risk routine cases use low delay, low congestion, few or no malfunctions, adequate trolley availability, and confirmed or absent assistance requests. Maintenance-heavy cases stress malfunction count and severity. Inventory-heavy cases stress trolley availability and shortage indicators. Congestion/delay-heavy cases stress arrival delay and gate congestion. Assistance-readiness cases vary assistance request status and confirmation. Composite multi-risk cases combine multiple stressors.

Figure 3 illustrates the resulting readiness distribution, highlighting the spread of outcomes across the synthetic scenarios. Figure 4 further breaks down these outcomes by alert level, visualizing the count distribution across Routine, Watch, Priority, and Critical categories. For each generated inbound case, the evaluation records readiness score, alert level, dominant bottleneck, module-level risks, action-queue size, priority-action counts, and top action categories. The sweep is a controlled architecture-behavior evaluation. It does not replace the seeded demonstration cases and does not create observational airline evidence.

**Figure 3:**
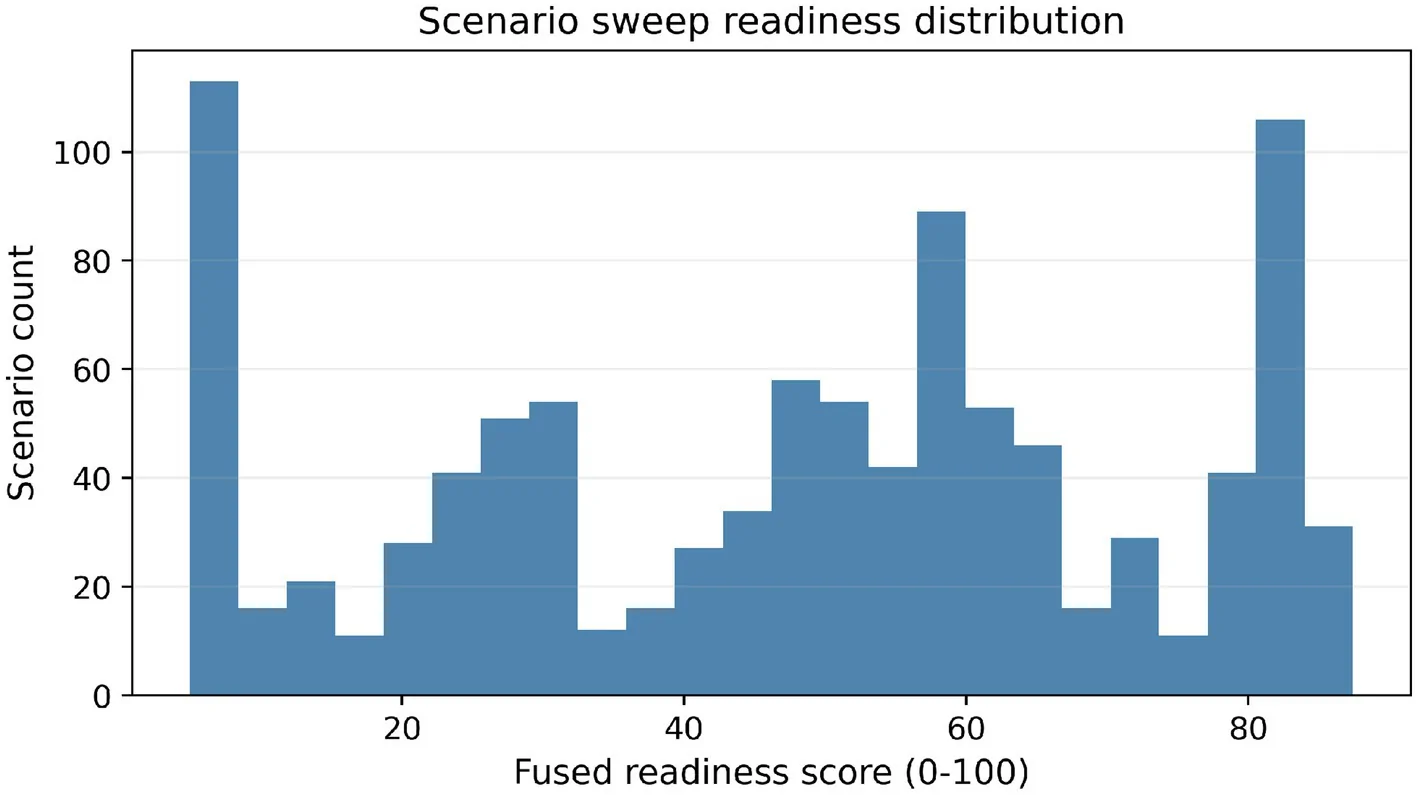
Readiness distribution across the controlled synthetic scenario sweep.

**Figure 4:**
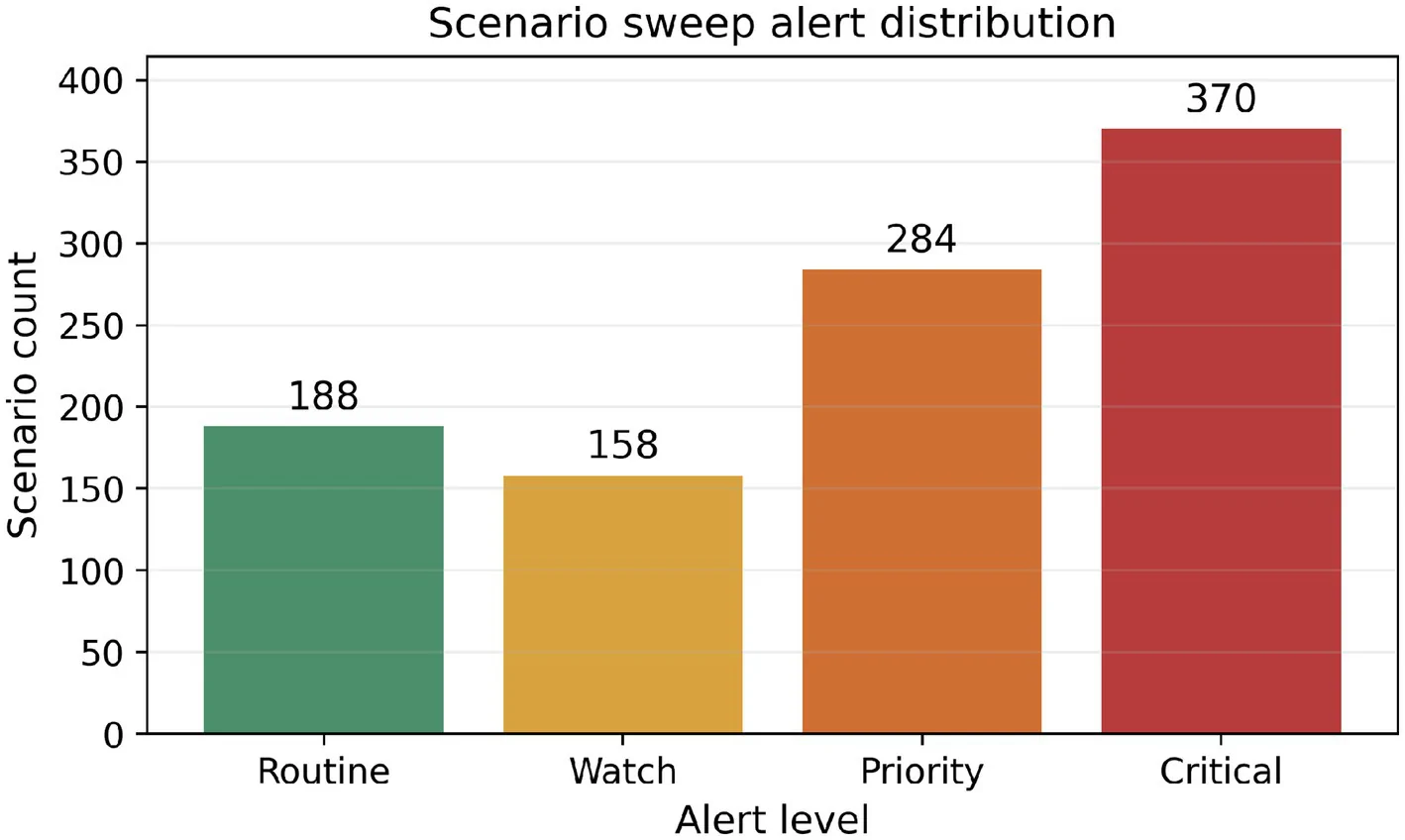
Alert-level distribution across the controlled synthetic scenario sweep.

## 7 Regime-level results

The generated regimes produced differentiated system behavior. The low-risk routine regime had mean readiness 82.34, while the maintenance-heavy regime had mean readiness 26.17, the inventory-heavy regime had mean readiness 54.62, and the composite multi-risk regime had mean readiness 8.77. These values should be interpreted as designed scenario-response behavior, not as observed airline probabilities.

Table 3 aggregates the regime-level performance, contrasting mean and median readiness scores across the six operational regimes, while Figure 5 visualizes these performance distributions, illustrating how different stressor combinations create distinct readiness profiles that differentiate the system’s operational responses.

**Table 3:** Regime-level summary of synthetic scenario responses.

Regime	*n*	Mean readiness	Median readiness	Dominant bottleneck	Maint. risk	Inv. risk	Assist. effect
Assistance	166	66.58	65.50	Boarding	0.247	0.302	8.63
Composite	166	8.77	5.00	Maintenance	0.784	0.872	4.80
Congestion/delay	167	45.78	46.40	Boarding	0.287	0.758	6.54
Inventory	167	54.62	56.20	Catering	0.288	0.869	7.23
Routine	167	82.34	82.40	Cleaning	0.151	0.200	N/A
Maintenance	167	26.17	26.60	Maintenance	0.884	0.365	9.31

**Figure 5:**
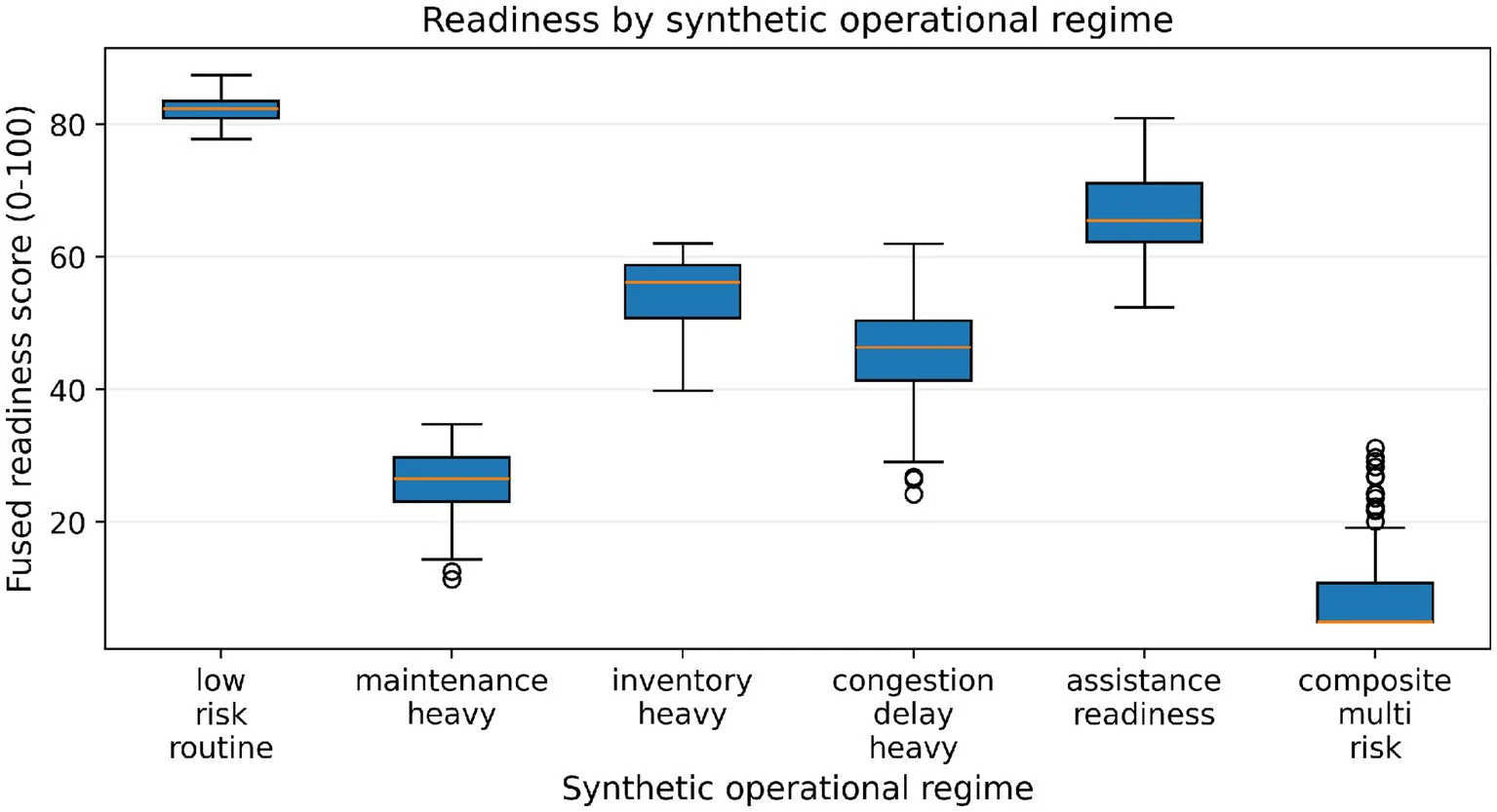
Readiness by synthetic operational regime.

## 8 Monotonicity and sanity checks

The monotonic checks test whether the system behaves coherently when one stressor changes while other inputs are held constant. Six of eight checks passed under a strict zero-tolerance criterion. Inventory shortage, trolley availability, arrival delay, gate congestion, assistance readiness, and composite-versus-low-risk checks behaved in the expected direction. The malfunction-severity and malfunction-count checks did not pass strictly: readiness rose by at most 0.4 and 0.5 points, respectively, on the 0–100 scale, at a single interior grid point in the flat region before the maintenance urgency class crossed from OK to PLAN, after which readiness fell correctly by 17.6 and 16.8 points. Maintenance failure probability increased monotonically with the stressor in both cases. Because readiness is dominated by discrete risk-category thresholds, this residual sub-threshold variation means that under any operational-indifference tolerance of half a readiness point or more, all eight sweeps are monotonic.

We report these strict-tolerance violations transparently rather than relaxing the threshold to mask them. They show that the trained-module-plus-fusion response is threshold-driven rather than smoothly monotonic, so a deployment-oriented study would still add monotonic post-model guards, calibrated fusion constraints, or expert-reviewed thresholds. Their magnitude, however, is operationally negligible relative to the category-level readiness changes the system is designed to signal, and they are not positive field evidence.

Table 4 reports the results of the monotonicity and sanity checks, confirming the system’s coherent behavior under varying stressors, and Figure 6 presents the corresponding sensitivity curves, which provide a granular view of how individual stressors, such as malfunction severity or trolley availability, influence the system’s fused readiness output.

**Table 4:** Monotonicity and sanity-check summary.

Test	Passed	Criterion
Malfunction severity	No	Failure risk and urgency non-decreasing; readiness non-increasing
Malfunction count	No	Readiness non-increasing
Inventory shortage score	Yes	Inventory risk non-decreasing
Trolley availability	Yes	Inventory risk non-decreasing as availability decreases
Arrival delay	Yes	Readiness non-increasing
Gate congestion	Yes	Readiness non-increasing
Assistance request	Yes	Unconfirmed assistance request does not improve readiness relative to no request or confirmed support
Composite vs. low risk	Yes	Composite multi-risk case has lower readiness than isolated low-risk case

**Figure 6:**
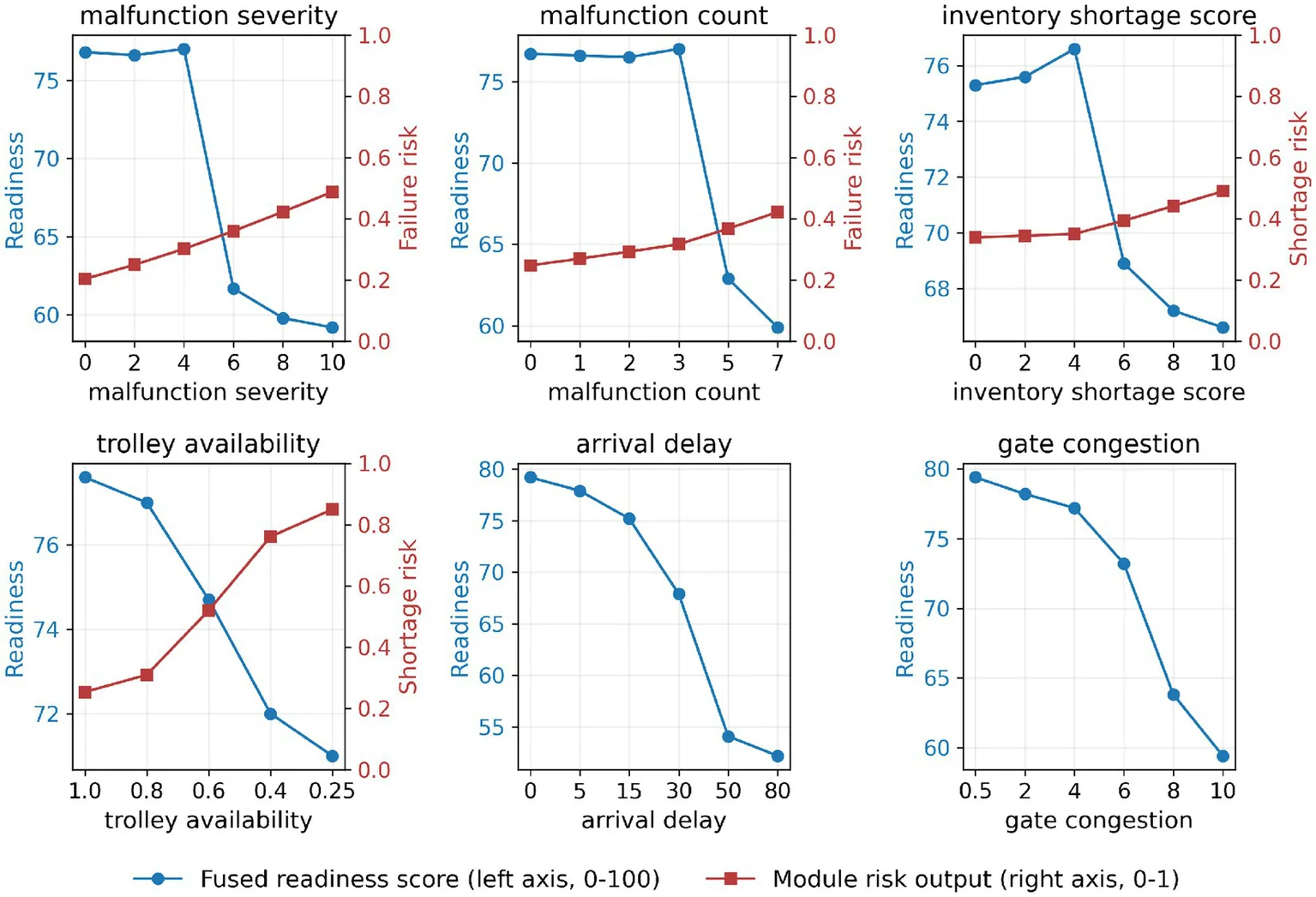
Sensitivity curves for one-factor system-behavior checks.

A deployment-oriented version of this layer would make the monotonicity property explicit rather than emergent. Concretely, a post-model guard would wrap the fusion output with hard constraints of the form readiness must be non-increasing in malfunction severity, malfunction count, inventory shortage, arrival delay, and gate congestion, holding other inputs fixed, enforced either by projecting any violating output back onto the nearest monotone value, by isotonic post-processing of the readiness curve along each stressor axis, or by constraining the fusion to a monotone functional form in the first place. The same guard could assert class-level invariants—for example that an escalation in maintenance urgency can never reduce the alert level—which are cheaper to verify and easier for an operator to trust than pointwise numerical monotonicity.

Such guards are not free. Strict monotonicity in every input forbids legitimate non-monotone interactions: a moderate stressor that triggers an earlier, better-resourced intervention may genuinely leave an aircraft better prepared than a marginally milder case that stays below an action threshold, and a strictly monotone layer would be unable to represent that. Enforcing monotonicity also flattens the threshold structure that makes the current output legible to operators, since it pushes the system toward smooth responses where operational practice is organized around discrete categories. The deviations reported above are sub-threshold, at most 0.5 readiness points, and arise precisely at this boundary between smooth penalty accumulation and discrete category change. We therefore treat the choice as a genuine design trade-off to be settled with operational evidence—strict guards buy verifiability and operator trust at the cost of expressiveness—rather than as a defect with a single correct fix.

## 9 Ablation and baseline results

The ablation study compares the full fusion system against configurations in which maintenance, inventory, bottleneck, or assistance influence is neutralized. The objective is not to prove real operational superiority. The objective is to show whether each signal family changes the behavior of the integrated action logic under controlled synthetic scenarios. Table 5 details the impact of removing specific signal families on the system’s overall performance, and Figure 7 highlights these findings by visualizing the delta in mean readiness for each ablation; further, Table 6 compares the full integrated system against various single-signal baselines, demonstrating the architecture’s capacity for cross-domain action differentiation.

**Table 5:** Ablation summary relative to the full integrated system.

Configuration	Mean readiness	Delta from full	Critical	Priority	Mean actions	Top action changes
Full system	47.40	0.00	370	284	4.60	0
No assistance in fusion	48.83	1.43	362	284	4.42	108
No bottleneck in fusion	57.39	9.99	208	238	4.66	122
No inventory in fusion	54.13	6.73	330	91	4.07	187
No maintenance in fusion	61.05	13.65	79	307	3.94	319

**Figure 7:**
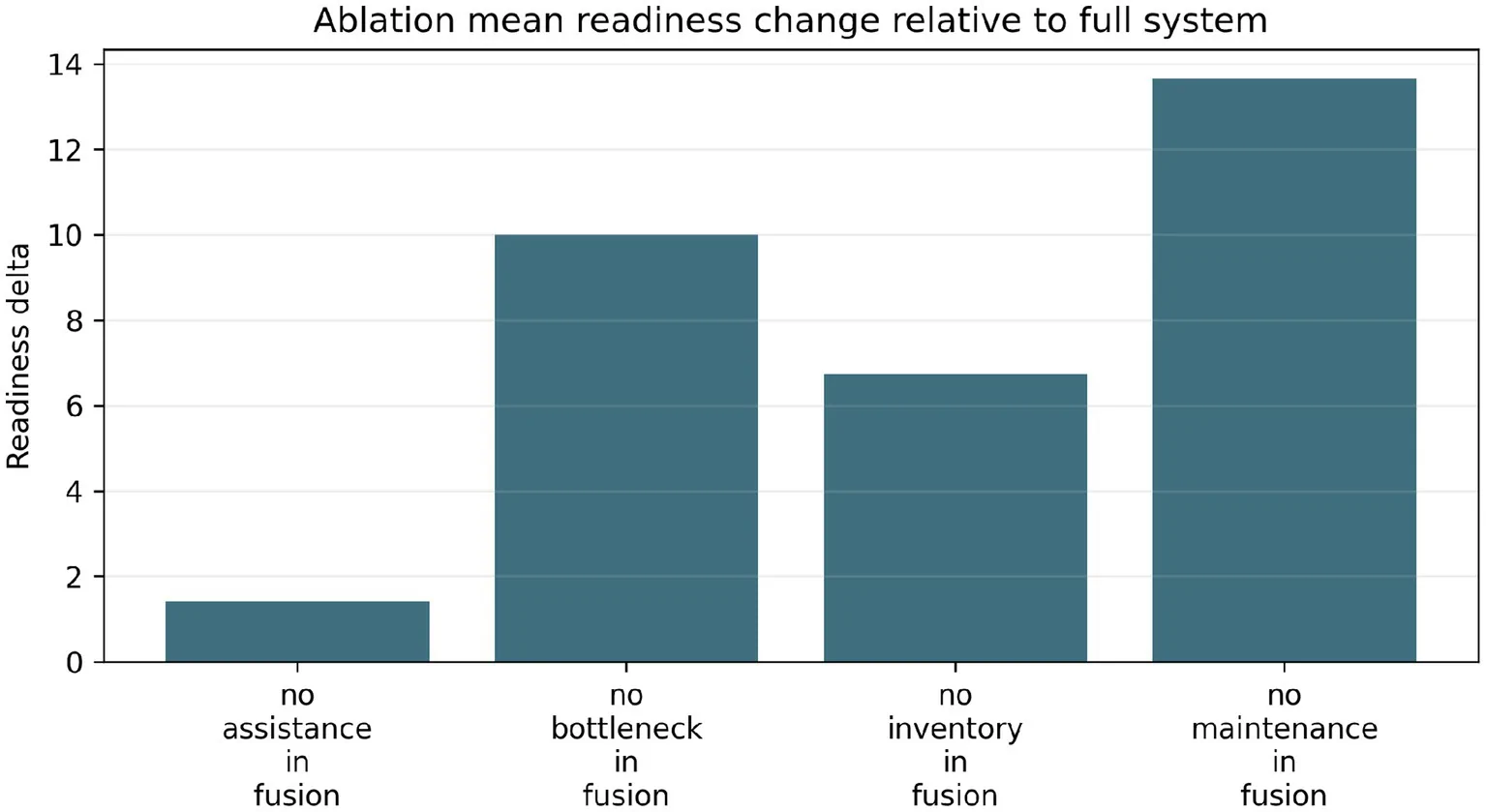
Mean readiness change when signal families are removed from fusion.

**Table 6:** Baseline comparison for controlled synthetic behavior differentiation.

Baseline	Mean readiness	Std. readiness	Alert levels	Dominant signals	Action categories	Critical	Priority
Static delay/gate baseline	73.44	13.65	3	1	1	0	168
Maintenance-only baseline	73.56	23.76	4	4	2	13	306
Inventory-only baseline	69.15	15.80	3	3	2	0	401
Bottleneck-only baseline	50.18	13.13	4	5	5	201	465
Full integrated OCC fusion	47.40	25.04	4	5	7	370	284

The full configuration uses maintenance, inventory, bottleneck, assistance, and fusion logic together. The no-maintenance ablation neutralizes maintenance influence in the fusion layer. The no-inventory ablation neutralizes inventory-shortage influence. The no-bottleneck ablation replaces process-class bottleneck evidence with a neutral bottleneck state. The no-assistance ablation removes assistance-readiness influence. Each configuration is evaluated on the same generated scenario inputs, so differences reflect fusion behavior rather than different scenario samples.

The baseline comparison contrasts the full system with static delay/gate, maintenance-only, inventory-only, and bottleneck-only baselines. The static delay/gate baseline uses only arrival delay and gate congestion; each single-signal baseline exposes one module family at a time and reuses that module’s own output. The full system produces more cross-domain action differentiation than the single-signal baselines, with more distinct alert levels, dominant signals, and action categories. Because no ground-truth operational outcome is available, this demonstrates that the fusion integrates heterogeneous signals into differentiated advisory outputs—an architectural property—rather than that its individual decisions are more accurate or operationally superior.

To confirm that these differences are not artifacts of the particular 1,000-case sample, we computed 2000-resample bootstrap 95% confidence intervals. The six regime mean-readiness values are cleanly separated with non-overlapping intervals, from low-risk routine (82.3, 95% CI [82.1, 82.6]) down to composite multi-risk (8.8, [7.9, 9.7]). The ablation effects are likewise stable and rank-ordered: mean readiness rises by 13.7 points ([12.6, 14.7]) when maintenance influence is removed, 10.0 ([9.7, 10.3]) for bottleneck, 6.7 ([6.5, 7.0]) for inventory, and 1.4 ([1.2, 1.6]) for assistance. The differentiation is therefore stable to resampling within the synthetic population. A stronger multi-seed check that regenerates five independent 1,000-case populations under different random seeds confirms it: each regime mean varies by less than half a point in standard deviation across seeds, and the maintenance > bottleneck > inventory > assistance ablation ordering is unchanged (full per-metric values are in the reproducibility archive). The differentiation is thus a stable property of the architecture rather than an artifact of one synthetic draw. Sensitivity to the generator’s deeper design choices, such as alert thresholds and regime proportions, remains a separate question that real operational data and an operator study would settle.

## 10 Operator interface

The interface remains a supporting artifact, not the main evidence source. It shows how readiness, alerts, provenance, bottleneck state, and action queues can be presented for operator review. A single representative view is retained in the main paper to avoid making the manuscript look like a software demo report. Figure 8 displays that view of the OCC dashboard, showing how the integrated readiness scores, alert levels, and action queues are surfaced for operator review in a research-demonstrator context. Because the full dashboard cannot be reproduced at a legible type size within the page width, Figure 9 enlarges the inbound flight table from the same view, divided into two stacked strips that repeat the flight identifier column, so that the readiness values, alert levels, dominant bottleneck, and per-domain maintenance, inventory, and assistance indicators can each be read directly.

**Figure 8:**
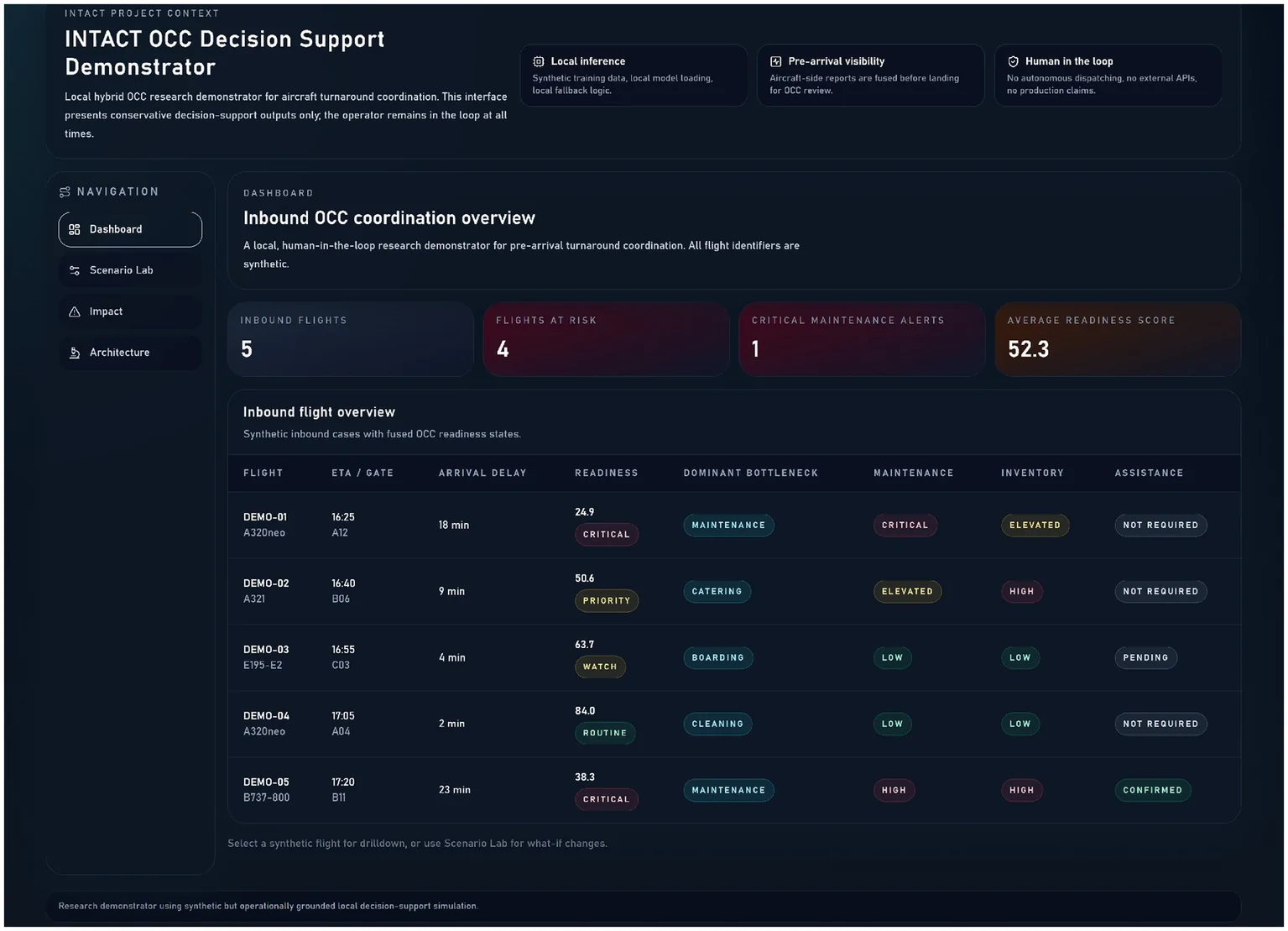
Representative OCC dashboard view showing fused pre-arrival summaries. The complete operator view is shown; Figure 9 enlarges the inbound flight table.

**Figure 9:**
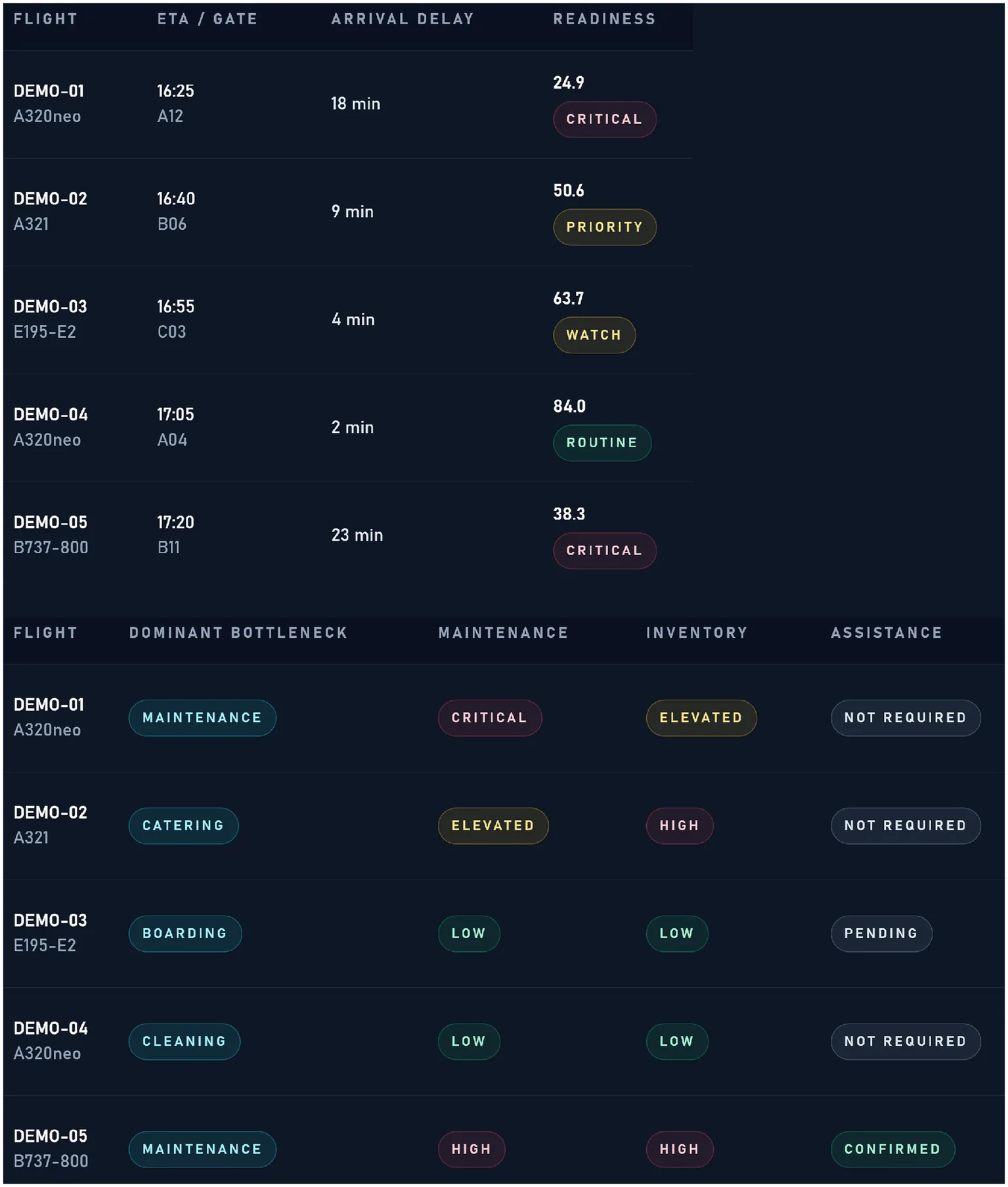
Inbound flight table from the Figure 8 dashboard view, enlarged and divided into two stacked strips. The upper strip shows flight identity, estimated time of arrival and gate, arrival delay, and fused readiness with its alert level; the lower strip repeats the flight identifier and shows the dominant predicted bottleneck together with the per-domain maintenance, inventory, and assistance-readiness indicators. All values are synthetic scenario outputs.

### 10.1 Human factors and interaction design

The human-in-the-loop claim in this paper is an architectural commitment—the system issues advisory outputs that require operator action and never dispatches autonomously—and we make explicit here what that commitment does and does not currently cover, since interaction design is where a decision-support layer most often fails in practice. Three interaction questions are consequential and are not settled by the present evidence base. The first is alert volume. The scenario sweep produced 370 Critical and 284 Priority cases out of 1,000, but this is a property of a deliberately stressed synthetic population and must not be read as an expected operational alert rate; in a real OCC the sustainable rate is bounded by operator attention, and an advisory layer that exceeds it degrades into alarm flooding, which is a well-documented failure mode of safety-critical alerting. A deployed configuration would therefore need its thresholds calibrated to a target alert rate per operator per shift, established empirically, rather than to the nominal thresholds used here. The second is prioritization under simultaneous criticality. The present design orders the action queue by a four-level priority and surfaces the dominant bottleneck per aircraft, but it does not arbitrate across aircraft: when several inbound flights are Critical at once, the interface presents them in parallel and leaves the cross-aircraft trade-off—which is a resource and network decision, not a readiness computation—to the operator.

Making that arbitration explicit, for example by ranking on downstream rotation impact, is a substantive design extension and would require the network context the demonstrator does not model. The third is operator feedback. The current interface is one-directional: it presents readiness, provenance, and recommended actions, but provides no mechanism for an operator to mark a recommendation as inappropriate, attach a contextual note, or record the action actually taken. We regard this as the most important interface gap, both because such a channel is the precondition for the learning loop described in Section 11.5 and because a system that cannot be contradicted by its user invites the over-reliance discussed in Section 11.4. These are design requirements that follow from the architecture, not results. Establishing sustainable alert rates, cross-aircraft prioritization logic, and feedback affordances requires an OCC user study with operational staff, which remains, with real turnaround data, the decisive next step for this work.

## 11 Discussion

### 11.1 Principal findings and design implications

The results support the architectural claim that heterogeneous pre-arrival signals can be fused into differentiated OCC advisory outputs. Maintenance-heavy, inventory-heavy, congestion-heavy, assistance-readiness, routine, and composite scenarios lead to distinct readiness states, alert distributions, bottleneck profiles, and action categories. This is the appropriate contribution level for the current evidence base.

For air transport management readers, the practical implication is not that the demonstrator is ready for airline use. The implication is that pre-arrival information can be structured into a transparent workflow that helps reason about cross-domain turnaround readiness before arrival. A maintenance issue can change stand-side preparation, an inventory issue can affect catering and boarding readiness, and an unconfirmed assistance request can reduce coordination margin even when other modules are nominal.

The ablation ordering gives a concrete, if bounded, design signal: maintenance and bottleneck evidence move fused readiness and the selected operator action the most, while inventory and assistance act as smaller modifiers. For OCC practice, this suggests that pre-arrival attention and data-quality effort are most leveraged on maintenance-risk and process-bottleneck signals, with inventory and assistance handled as readiness modifiers rather than primary triggers. This is a hypothesis generated under synthetic conditions and is offered to guide, not replace, evaluation on real operations.

### 11.2 Emergent behavior and the complexity of failure

The architecture presented here is an intervention into a coordination system, and complexity-informed safety science cautions against evaluating such an intervention purely by its intended local effect. Dekker et al. (2011) argue that in complex systems failure is an emergent product of interactions rather than a linear chain of component faults, so that the meaningful question about a new layer is not only whether each module is accurate but how the layer changes the pattern of interactions around it. Our evaluation, being a controlled synthetic sweep of independent cases, is structurally incapable of observing such emergence; we therefore state the expected mechanisms explicitly so they can be tested rather than assumed away.

At least three are foreseeable. First, the ablation ordering reported in Section 9—maintenance and bottleneck signals dominating the change in fused readiness—is a statement about the fusion layer, but if it were mirrored in operator behavior it would amount to a systematic reallocation of pre-arrival attention toward maintenance-heavy flights. The corresponding risk is displacement: attention and scarce ground resources drawn toward scored maintenance risk may create new vulnerabilities in exactly the domains the architecture scores more weakly, such as passenger handling, assistance provision, and gate allocation. Second, the threshold structure documented in Section 8 is a coordination signal as well as a number. Because alert levels change discretely, cases clustered near a boundary can be treated categorically differently despite near-identical underlying states, and under organizational pressure such boundaries tend to acquire procedural force—an aircraft that is “Critical” may attract resources that an aircraft one readiness point above the threshold does not, irrespective of operational merit. Third, the fusion layer itself can become a source of systemic resonance: because every inbound aircraft in an OCC view is scored by the same rule set with the same thresholds, a common-mode shift—a mis-specified coefficient, a drifting upstream module, or a systematic scenario bias—would move many aircraft in the same direction at the same time, converting an independent per-aircraft advisory into a correlated, fleet-wide misdirection of attention. This is the mechanism by which a decision-support layer contributes to, rather than mitigates, emergent failure, and it argues for monitoring the distribution of system outputs over time, not only individual recommendations.

### 11.3 Organizational preconditions, roles, and safety culture

Komljenovic et al. (2017) argue that in complex operational and business environments safety improvement is primarily an organizational rather than a technical achievement, because organizational structures, incentives, and communication patterns determine whether a technical capability is used as intended. The present study evaluates architecture and scenario behavior and is silent on this dimension; we set out here what the architecture would require of a receiving organization, as a set of preconditions rather than claims.

Introducing readiness scores and action queues changes the distribution of authority in an OCC. An action queue that names a team and a priority is an implicit allocation of responsibility, and unless the accountability for acting, for declining to act, and for overriding is defined in advance, the system can produce diffusion of responsibility precisely in the compounded cases it is designed to flag. Four organizational preconditions follow. Training must convey not only the interface but the evidence boundary: operators should know that the scores are heuristic fusions of module outputs, that the coefficients are not calibrated against operational outcomes, and where the system is known to be weak. Governance must define escalation and override explicitly—who may act on a P1 item, who may set it aside, and how an override is recorded—so that overriding the system is a legitimate, documented act rather than a deviation. Integration with existing Safety Management Systems must be deliberate: readiness states and alert histories are candidate inputs to risk registers and occurrence reporting, but if the advisory layer is maintained as a parallel channel it will create a new silo and compete with established reporting rather than enrich it. Finally, the organization must anticipate adaptation and resistance as normal rather than aberrant: operators who find the alert rate unsustainable will rationally learn to discount categories of alert, and that adaptation, if unobserved, silently removes the safety benefit the system was introduced to provide.

### 11.4 Complacency, risk perception, and the illusion of control

Årstad and Aven (2017) warn that in major-accident contexts complacency and complexity combine to produce an illusion of control, in which the visible, quantified management of risk generates confidence while un-quantified uncertainties remain. A pre-arrival readiness score is exactly the kind of artifact that can produce this effect, and the explicit evidence boundaries maintained throughout this paper, while a genuine safeguard at the point of publication, offer no protection once a number is rendered on an operational display.

Three mechanisms deserve naming. The first is misplaced confidence in the score itself: a readiness value of 84.0 carries an implied precision that the underlying heuristic penalties do not possess, and a single displayed number invites interpretation as a measurement rather than an aggregation of assumptions. The second is attentional displacement toward the quantified. The architecture scores maintenance, inventory, bottleneck, congestion, delay, and assistance-confirmation status; it does not score crew fatigue, organizational strain, informal coordination between ground teams, local workarounds, or the experience of the individual duty manager. A system that renders the first set numerically and the second not at all will, by construction, bias attention toward what it can count, and the danger is greatest precisely when the unscored factors dominate. The third is authority creep under time pressure: an advisory output consulted under compressed turnaround timing tends to be treated as an answer rather than an input, and the more consistent and confident the interface appears, the stronger that tendency.

Countermeasures are organizational and are stated here as requirements for any deployment. Training should foreground uncertainty and residual risk rather than interface mechanics, and should include cases where the system is wrong. The interface should preserve the provenance and readiness-breakdown affordances already in the design, since a score that can be decomposed is harder to treat as an oracle than one that cannot; a deployed version should additionally communicate uncertainty rather than a point value. Most importantly, use should be audited: monitoring override rates, the correspondence between recommended and actual actions, and the drift of both over time is the only practical way to detect movement toward either over-reliance or wholesale disregard, both of which are failures, and neither of which is visible from the system’s own outputs.

### 11.5 Supporting safety investigation and organizational learning

A consequence of the complexity perspective is that investigation after a disruption should examine systemic interactions and performance variability rather than isolate a single proximate cause (Dekker et al., 2011). The architecture has an unexploited affordance here, which we note as a direction rather than a result: it generates a timestamped, decomposable record of how a situation appeared before it developed.

Because readiness scores, alert levels, provenance labels, and action queues are produced pre-arrival and can be logged, they permit reconstruction of the decision landscape as it stood at the time, rather than as it is remembered afterwards—directly addressing the hindsight and time-reversibility problems that complexity-informed investigation identifies in conventional accident analysis. Three uses follow. Logged advisory state supports reconstruction of what information was available and how it was prioritized at each point before a disruption.

Systematic comparison of recommended against actually-taken actions characterizes organizational adaptation, resistance, and improvisation as observable patterns instead of individual deviations, which is a precondition for non-blaming, Safety-II-oriented learning about how OCC performance is actually produced under stress. And the scenario-sweep machinery reported here can be repurposed for counterfactual analysis in investigation: the same deterministic pipeline that generated the sweep can replay a real disruption under varied inputs to ask which changes would, and would not, have altered the advisory state. Realizing any of this requires the operator-feedback channel identified as missing in Section 10.1, together with a governance agreement that such logs are used for learning rather than for individual performance assessment—a condition that is organizational, not technical, and without which the same records would suppress the candor the approach depends on.

## 12 Limitations and threats to validity

The main limitation is synthetic data. The scenario sweep is controlled and reproducible, but it is not observational airline evidence. The system has not been evaluated on real airline, airport, or station-operation data. No OCC user study has been conducted. No airline deployment, certification, safety assurance, operational savings, or return-on-investment claim is made. The readiness thresholds are heuristic. The fusion layer is transparent but rule-based rather than learned from OCC decisions. The maintenance and inventory modules are trained on synthetic labels. The bottleneck model predicts process classes, not full turnaround duration. Passenger assistance is readiness logic, not localization ML.

Because the reported distributions are generated rather than observed, every frequency in this paper is an artifact of the generator’s design and must not be read as an operational rate. The regime proportions, the 370 Critical, 284 Priority, and 188 Routine alert counts, the mean readiness of 47.4, the per-regime means, and the dominant-bottleneck counts, for example the maintenance bottleneck count of 319, all follow directly from the chosen regime mix, stressor ranges, and thresholds; a different generator specification would produce different values with no change to the architecture. They are properties of a deliberately stressed test population, constructed to exercise the fusion across its operating range, and they carry no information about how often such states occur in an airline’s actual traffic.

This has a design consequence that we state plainly. Because the same operational assumptions inform both the scenario generator and the modules being evaluated, synthetic evaluation can quietly validate the assumptions it was built on, and iterating design decisions—threshold placement, penalty magnitudes, regime definitions—against such a population risks tuning the system to its own generator rather than to operations. The ablation ordering we report should therefore be read as a statement about signal influence inside the fusion, not as evidence about which signals matter most in practice. Guarding against this requires confrontation with real turnaround records, and, because the questions raised in Sections 11.3 and 11.4 concern how the system is used rather than how it computes, ethnographic and organizational study of OCC practice alongside quantitative validation.

The two strict-tolerance monotonicity deviations are sub-threshold, at most half a readiness point, but still indicate that a deployment-oriented study would add monotonic guards or calibrated fusion constraints. More fundamentally, because the trained modules are fit to synthetic labels and the scenarios are generated from related operational assumptions, the evaluation characterizes internal architectural behavior and cannot, by construction, establish predictive accuracy or operational value; the ablation magnitudes quantify signal influence within the fusion, not validated importance. The decisive next step is therefore not more synthetic analysis but confrontation with real turnaround records and an OCC user study, which would confirm or falsify whether the differentiated readiness states correspond to operationally meaningful distinctions.

## 13 Conclusion

This paper presents a pre-arrival OCC decision-support architecture for aircraft turnaround coordination and evaluates it through controlled synthetic scenarios. The results show that heterogeneous pre-arrival maintenance, inventory, bottleneck, and assistance-readiness signals can be fused into differentiated advisory outputs while preserving a transparent human-in-the-loop workflow. The study also shows the limits of the present evidence base: controlled scenario behavior is useful for architecture evaluation, but it is not a substitute for airline data, expert review, OCC user evaluation, or deployment-oriented validation. Looking forward, the reproducible architecture and scenario-evaluation package provided here offer a baseline for future research. By demonstrating how fragmented pre-arrival signals can be organized for transparent OCC review, this work creates a foundation for subsequent studies that incorporate real-world airline data, high-fidelity operator-in-the-loop experiments, and safety-assurance frameworks, ultimately helping the industry move toward more structured, data-informed pre-arrival coordination.

## Data Availability

The datasets presented in this study can be found in online repositories. The names of the repository/repositories and accession number(s) can be found below: https://github.com/purohit0208/intact-occ-decision-support-demonstrator.
